# Inhibitory Potential and Binding Thermodynamics of Scyllatoxin‐Based BH3 Domain Mimetics Targeting Repressor BCL2 Proteins

**DOI:** 10.1002/jmr.70001

**Published:** 2025-02-04

**Authors:** H. A. D. B. Amarasiri, Danushka Arachchige, Matthew J. K. Vince, Justin M. Holub

**Affiliations:** ^1^ Department of Chemistry and Biochemistry Ohio University Athens Ohio USA; ^2^ Institut für Bioanalytische Chemie, Biotechnologisch‐Biomedizinisches Zentrum, Fakultät für Chemie Und Mineralogie Universität Leipzig Leipzig Germany; ^3^ Molecular and Cellular Biology Program Ohio University Athens Ohio USA; ^4^ Edison Biotechnology Institute Ohio University Athens Ohio USA

**Keywords:** BH3 domain, binding thermodynamics, competitive binding, disulfide linkage, effector BCL2 proteins, peptide mimetic, protein–protein interaction, repressor BCL2 proteins, scyllatoxin

## Abstract

The B‐cell lymphoma 2 (BCL2) proteins are a class of apoptosis regulators that control the release of apoptogenic factors from mitochondria. Under normal physiological conditions, apoptosis is inhibited through the actions of anti‐apoptotic (repressor) BCL2 proteins that bind semi‐indiscriminately to the helical BH3 domains of pro‐apoptotic (effector) BCL2 proteins. In this work, we developed a series of BH3 domain mimetics by grafting residues from the effector BCL2 protein Bax onto the α‐helix of scyllatoxin (ScTx). These so‐called “ScTx‐Bax” constructs were then used to gain insight into the physicochemical nature of repressor/effector BCL2 interactions. Specifically, we utilized competitive binding and isothermal titration calorimetry (ITC) to investigate the inhibitory potential and binding thermodynamics of ScTx‐Bax structural variants that target the repressor protein Bcl‐2 (proper) in vitro. Our data show that ScTx‐Bax mimetics compete with isolated Bax BH3 domain peptides for Bcl‐2 with IC_50_ values in the mid‐nanomolar range and that greater flexibility within the ScTx‐Bax BH3 domain correlates with more effective inhibition. Furthermore, ITC experiments revealed that unstructured ScTx‐Bax variants target Bcl‐2 with greater entropic, but lower enthalpic, efficiencies than structured ScTx‐Bax peptides. These results suggest that entropic contributions to binding Bcl‐2 are more favorable for flexible BH3 domains; however, this enhancement is counterbalanced by a moderate enthalpic penalty. Overall, this study improves understanding of how structural properties of effector BH3 domains influence the promiscuous binding patterns of BCL2 proteins and expands the utility of ScTx‐based BH3 domain mimetics as molecular tools to study discrete recognition elements that facilitate repressor/effector BCL2 interactions.

**Abbreviations**
5‐CF: 5‐carboxyfluorescein
ACN: acetonitrile
B‐PER: bacterial protein extraction reagent
DCM: dichloromethane
DIEA: N,N‐diisopropylethylamine
HATU: O‐(7‐azabenzotriazol‐1‐yl)‐N,N,N′,N′‐tetramethyluronium hexafluorophosphate
HCTU: 2‐(6‐chloro‐1‐H‐benzotriazole‐1‐yl)‐1,1,3,3‐tetramethylaminium hexafluorophosphate
IPTG: isopropyl β‐D‐1‐thiogalactopyranoside
MMP: 4‐methylmorpholine
NMP: N‐methyl‐2‐pyrrolidone
SDS: sodium dodecyl sulfate
TFA: trifluoroacetic acid
TFE: 2,2,2‐trifluoroethanol
TIPS: triisopropylsilane

## 1 Introduction

Signaling cascades along the intrinsic apoptosis pathway are controlled, in part, by proteins in the B‐cell lymphoma 2 (BCL2) family. BCL2 proteins are associated with the outer mitochondrial membrane, where they play important roles in regulating the release of apoptogenic factors into the cytoplasm [1, 2]. There are 25 known genes in the BCL2 family [3], each of which are classified as either repressors, effectors, or activators based on their role in regulating apoptosis. For example, repressor BCL2 proteins, which include Bcl‐2 (proper) and Bcl‐X_L_, have anti‐apoptotic (pro‐survival) activity and function by sequestering effector BCL2 proteins within the outer mitochondrial membrane [4]. Effector BCL2 proteins, such as Bcl‐2‐associated X protein (Bax) and Bcl‐2 homologous agonist/killer (Bak), have pro‐apoptotic (pro‐death) activity and oligomerize into pore‐forming units on the outer mitochondrial membrane upon activation [5]. Structurally, effector BCL2 proteins contain a semi‐conserved helical BCL2 homology 3 (BH3) domain that binds a shallow hydrophobic groove on the surface of repressor BCL2 proteins [6]. Under normal physiological conditions, effector BCL2 proteins remain inactive while bound to their cognate repressors and become activated only upon disruption of the BH3:BCL2 interaction. During times of cellular stress, activator BCL2 proteins such as Bcl‐2‐like protein 11 (Bim) and the BH3 interacting‐domain death agonist (Bid) are upregulated and compete with effectors for the BH3‐binding site on repressor BCL2 proteins [7]. This competitive binding event frees effector proteins from repressor‐induced sequestration, allowing them to facilitate release of cytochrome c and other apoptogenic factors from the mitochondria.

The concerted interactions between repressor, effector and activator BCL2 proteins represent critical regulatory events that ultimately determine cell fate. In general, healthy cells are allowed to thrive and propagate because apoptosis is restrained by repressor BCL2 proteins thorough their dynamic associations with cognate effectors. Conversely, dysregulated or aberrant activity among BCL2 proteins can lead to the collapse of natural apoptotic signaling pathways and the onset of serious pathophysiological conditions. Indeed, overexpression of the repressor protein Bcl‐2 is linked to oncogenesis in certain tissues [8, 9] and the loss of the activator protein Bim is associated with the development of autoimmune disorders [10]. Owing to the significant role BCL2 proteins play in disease pathogenesis, many clinical research and drug development programs have focused heavily on developing modulators of BCL2 activity. Unfortunately, efforts to develop molecules that selectively inhibit discrete BH3:BCL2 interactions have been hindered by varying specificities and extensive crosstalk among BCL2 members [11, 12]. The promiscuous interaction profiles among BCL2 proteins are thought to arise from highly‐conserved effector/activator BH3 domains binding semi‐indiscriminately to the relatively shallow, hydrophobic BH3‐binding grooves of repressor BCL2 proteins [13]. Consequently, further insight into the physicochemical properties that affect BH3:BCL2 recognition is needed to develop highly efficacious modulators of specific BCL2 interactions.

In addition to their obvious therapeutic relevance, BH3:BCL2 interactions have become useful model systems for studying generalized properties of protein association. In fact, researchers have studied the interactions of repressor, effector and activator BCL2 proteins using diverse experimental techniques such as fluorescence polarization (FP) [14], hydrogen deuterium exchange (HDX) [15], Förster resonance energy transfer (FRET) [16], nuclear magnetic resonance (NMR) [17], isothermal titration calorimetry (ITC) [18], surface plasmon resonance (SPR) [19] and x‐ray crystallography [18, 20]. In many cases, synthetic peptides that mimic the sequence of effector or activator BH3 domains have been used to directly assess binding specificities among repressor BCL2 proteins in vitro. In general, isolated peptides derived from effector or activator BH3 domains are unstructured in solution, and only fold into helical architectures upon targeting the hydrophobic BH3‐binding grooves of repressor BCL2 members [21, 22]. Such large‐scale conformational changes are often accompanied by significant thermodynamic fluctuations within the system resulting from entropic or enthalpic penalties incurred during protein association [23]. Furthermore, unstructured peptides are susceptible to proteolytic degradation in biological environments, which often limits their applications in vivo [24]. To circumvent these issues, synthetic BH3 domain mimetics that fold into stable secondary or tertiary structures have been developed by grafting residues required for BCL2 recognition onto natural proteins or structured peptides [25, 26]. In this so‐called “protein grafting” strategy, native helical regions contained within natural proteins or peptides are replaced with select amino acids from BH3 domains. This technique has resulted in the development of several well‐ordered BH3 domain mimetics that can target discrete repressor BCL2 proteins with enhanced precision [26, 27, 28, 29, 30, 31]. Importantly, the modular nature of synthetic peptides affords exquisite control over the primary sequence and folded architecture of the final construct, each of which can be fine‐tuned to enhance affinity and specificity of pre‐organized helical BH3 domains.

As an early proof of concept, Schepartz et al. developed derivatives of avian pancreatic peptide (aPP) in which residues of the Bak BH3 domain were grafted onto the α‐helix of aPP [27]. One derivative, PPBH3‐1, bound Bcl‐2 with 100‐fold higher affinity than isolated (unstructured) Bak BH3 domains. The PPBH3‐1 peptide was subsequently evolved into aPP‐Bak derivatives that displayed 10‐fold enhancement in selectivity for Bcl‐2 over Bcl‐X_L_ [28]. Moreover, Walensky et al. have reported extensively on hydrocarbon‐stapled helical BH3 domain mimetics that target repressor BCL2 proteins in vitro and in vivo [30, 32, 33]. In a seminal example of their work, the stapled Bid BH3 domain mimetic SAHB_A_ was found to target Bcl‐2 with 7‐fold higher affinity than unstructured Bid BH3 peptides [31]. Taken together, these studies suggest that pre‐organizing otherwise unstructured BH3 domains into stabilized α‐helices serves to enhance their specificity and affinity to cognate repressor BCL2 proteins. On the other hand, many therapeutically‐relevant PPIs are facilitated by the concerted folding and binding of at least one inherently disordered binding partner ([34, 35]). Such disorder‐to‐order transitions upon binding are often required for efficient interactions, especially along large, shallow protein interfaces. Indeed, it has been postulated that PPIs involving short, inherently disordered peptides actually provide promising binding sites that lead to highly selective and potent interactions ([36, 37]). Under these circumstances, the loss of entropy upon binding a flexible peptide is likely countered by targeting large hydrophobic sidechains to distinct pockets through an induced‐fit binding mechanism [38] and small hydrophobic sidechains relieving surface waters that interact unfavorably with the binding surface [39]. Taken together, these findings suggest that flexibility upon binding can be advantageous when designing chemical modulators of PPIs that lack the well‐defined cavities of classic drug targets. In fact, the future development of highly specific, potent inhibitors of such interactions will likely require a critical understanding of how flexibility among the native binding partners affects the favorability of the interaction.

In an effort to better understand the molecular nature of discrete BH3:BCL2 interactions, our lab has synthesized a series of Bax BH3 domain mimetics based on scyllatoxin (ScTx). ScTx is a small, 31‐amino acid protein that folds into an α/β structural motif stabilized by three disulfide linkages between cysteine residues C3—C12, C8—C26 and C12—C28 [40]. We have previously generated ScTx‐based Bax BH3 domain mimetics that target repressor BCL2 proteins with low micromolar affinity in vitro by grafting amino acids from the Bax BH3 domain important for Bcl‐2 recognition onto the α‐helix of ScTx [29, 41]. In this context, ScTx is a useful tool to study molecular recognition because it is comprised of a sequence‐based structure that allows for the addition or removal of native disulfide bonds. Notably, this unique characteristic affords access to varying degrees of structural rigidity that can be optimized to suit the molecular interaction being studied. Our group has used ScTx‐based peptides to identify how specific properties of the BH3 helix, such as amino acid sequence and structural flexibility, influence BH3:BCL2 interactions [29]. It was determined from our previous studies that ScTx‐Bax mimetics containing two or three native disulfide linkages are too rigid to effectively interact with Bcl‐2 in vitro [29, 42]. On the other hand, ScTx‐Bax mimetics containing zero or one disulfide linkage are able to target Bcl‐2 with low micromolar affinity [41]. Collectively, these results support the notion that an induced‐fit binding mechanism is required for favorable BH3:BCL2 interactions.

While these preliminary studies were ultimately successful, they were conducted using FP direct binding assays and did not inform on the inhibitory potential or binding thermodynamics of ScTx‐Bax peptides that target Bcl‐2. We were therefore interested in determining whether ScTx‐Bax mimetics could compete with natural ligand for the BH3‐binding pocket of Bcl‐2 and how structural flexibility of the ScTx‐Bax BH3 helix affects thermodynamics of binding. In this work, we assess the inhibitory potential and binding thermodynamics of three ScTx‐Bax sequence variants using competitive binding assays and ITC. Results from our competitive binding experiments showed that ScTx‐Bax mimetics compete with native Bax BH3 domain peptides for the BH3‐binding pocket of Bcl‐2 in vitro. Additionally, ITC experiments provided useful information on the thermodynamics of binding, such as changes in Gibbs free energy (Δ*G*), enthalpy (Δ*H*) and entropy (Δ*S*), that are associated with ScTx‐Bax:Bcl‐2 interactions. Importantly, these results highlight the significant role conformational flexibility within the Bax BH3 binding domain plays when targeting Bcl‐2 and may lead to a better understanding of how disorder‐to‐order transitions influence the molecular nature of BH3:BCL2 interactions.

## 2 Materials and Methods

### 2.1 Peptide Synthesis

All peptides developed herein were generated using a modification of standard Fmoc‐based solid phase peptide synthesis (SPPS) methods as described previously [43, 44]. Briefly, oligopeptides were synthesized on Fmoc‐PAL‐AM resin on a 25 μmol scale that was based on the resin loading level. To facilitate synthesis, the resin was washed with fresh N‐methyl‐2‐pyrrolidone (NMP) following each iterative coupling and deprotection step described. Amide bonds were formed by treating the resin with 5 equivalents (eq) of Fmoc‐amino acid, 5 eq of O‐(7‐azabenzotriazol‐1‐yl)‐N,N,N′,N′‐tetramethyluronium hexafluorophosphate (HATU) and 10 eq of N,N‐diisopropylethylamine (DIEA) in NMP while stirring at ambient temperature for 15 min. N‐terminal Fmoc groups were removed by treating the resin at ambient temperature for 15 min with 25% (v/v) piperidine in NMP containing 5% (v/v) formic acid to inhibit aspartimide formation [45]. Sequential cycles of amino acid coupling and deprotection were performed until peptide oligomers of desired sequence were obtained. Following synthesis, peptides were acetylated at their N‐terminus by treating the resin twice with 6% (v/v) acetic anhydride and 6% (v/v) 4‐methylmorpholine in NMP for 15 min at ambient temperature. To generate fluorescently‐labeled constructs, resin‐bound peptides were Fmoc deprotected and transferred to a solution containing 5 eq of 5‐carboxyfluorescein (5‐CF), 5 eq of 2‐(6‐chloro‐1‐H‐benzotriazole‐1‐yl)‐1,1,3,3‐tetramethylaminium hexafluorophosphate (HCTU) and 7.5 eq of DIEA in NMP. The 5‐CF labeling reaction was allowed to stir in the dark at ambient temperature for 24 h. Following acetylation or fluorescent labeling, the resin was washed with fresh NMP and dichloromethane (DCM) and dried under reduced pressure to remove residual solvent.

### 2.2 Cleavage and Purification of Peptides

Following synthesis, resin‐bound peptides were globally deprotected and cleaved from the solid support by treating the resin with 3 mL of cleavage cocktail composed of 88% trifluoroacetic acid (TFA), 5% water, 5% phenol and 2% triisopropylsilane (TIPS) (v/v/v/v), and stirring at ambient temperature for 1 h. Following cleavage, the peptides were precipitated in cold diethyl ether, pelleted by centrifugation and resuspended in 15% (v/v) aqueous acetonitrile (ACN). This solution was then frozen and lyophilized to dryness. Following lyophilization, crude peptide powders were dissolved in a suitable volume of 15% (v/v) aqueous ACN and purified across a semi‐preparatory scale, reversed‐phase C18 column (Hichrom, 10 μm, 250 × 10 mm) using a ProStar HPLC system (Agilent). For each purification, 2 mL of crude peptide solution was loaded onto the column and eluted over 50 min with a linear gradient of 15%–65% solvent B over solvent A (1% ACN/min), where solvent A is 0.1% (v/v) TFA in water and solvent B is 0.1% (v/v) TFA in ACN. Absorbances were monitored at 214 nm and 280 nm to identify acetylated peptide products or at 214 nm and 450 nm to identify fluorescently‐labeled peptides. Product peaks were collected, combined, frozen and lyophilized to dryness before being subjected to oxidation reactions. For peptides not being subjected to oxidation, product peaks from HPLC purifications were combined and lyophilized twice. Purified peptide powders were then stored at −20°C protected from light.

### 2.3 Oxidation of ScTx‐Bax Peptides

Reduced ScTx‐Bax peptides containing two cysteine residues were dissolved at a final concentration of 100 μM in 5 mL phosphate buffer (86 mM NaH_2_PO_4_, 14 mM Na_2_HPO_4_, pH 6) and allowed to stir for 24 h at ambient temperature while compressed air was gently bubbled into the solution. The extent of the air oxidation reaction was monitored by removing a small amount of the solution and injecting it across an analytical‐scale, reversed‐phase C18 column (Thermo, 5 μm, 50 × 2.1 mm). For analysis, the sample was eluted over 27 min with a linear solvent gradient of 5%–65.75% solvent B over solvent A (2.25% ACN/min). Upon elution, product peaks were collected and analyzed using matrix‐assisted laser desorption/ionization mass spectrometry (MALDI‐MS). Following completion of the reaction, the full volume of the mixture was loaded onto a ProStar HPLC system (Agilent) and purified across a semi‐preparatory scale reversed‐phase C18 column (Hichrom, 10 μm, 250 × 10 mm) using a linear gradient of 15%–65% solvent B over solvent A in 50 min (1% ACN/min). Purified fractions were then combined, frozen, lyophilized twice and stored at −20°C until further use.

### 2.4 Peptide Characterization

All peptide purities were evaluated by reversed‐phase HPLC using an Agilent ProStar HPLC system. To assess purity, peptides (5 μM in ultrapure water) were analyzed across an analytical‐scale, reversed‐phase C18 column (Thermo, 5 μm, 50 × 2.1 mm) and eluted over 20 min with a linear gradient of 5%–95% solvent B over solvent A (4.5% ACN/min). All peptides were purified to > 95% as determined by product peak integration of analytical HPLC chromatograms. Analytical HPLC data were processed using OpenLab CDS ChemStation Software (Agilent) v1.06 and KaleidaGraph v4.5 (Synergy Software). Product mass identities were confirmed using MALDI‐MS.

### 2.5 Disulfide Bridge Assignment

To confirm the position of the disulfide bonds, fully‐oxidized ScTx‐Bax peptides (50 μg) were mixed with trypsin (5%, w/v) in 100 μL digestion buffer (100 mM Tris, 1 mM CaCl_2_, pH 7.8) and the reaction was allowed to incubate at 37°C for 2 h as previously described [41, 42, 46]. Following completion of the reaction, 100 μL of 50% (v/v) aqueous TFA was added to stop the proteolysis. The full volume of the solution was then loaded onto an analytical‐scale, reversed‐phase C18 column (Thermo, 5 μm, 50 × 2.1 mm) and eluted over 50 min with a linear gradient of 0%–50% solvent B over solvent A (1% ACN/min). All major peaks resolved by HPLC were collected and analyzed by MALDI‐MS to determine peptide identity. As a negative control, proteases were similarly incubated in digestion buffer without peptide for 2 h at 37°C to verify that no autolytic fragments were formed during the reaction.

### 2.6 Circular Dichroism Spectropolarimetry

The structures of all peptides used in this work were evaluated in solution using wavelength‐dependent circular dichroism (CD) spectropolarimetry. For CD analysis, stock peptides were diluted to a final concentration of 10 μM in binding buffer (50 mM Tris, 100 mM NaCl, pH 8.0) supplemented with or without 30% 2,2,2‐trifluoroethanol (TFE). All peptide solutions were allowed to equilibrate at 20°C for 10 min before being analyzed. Far‐UV scans were performed from 250 nm to 190 nm on a Jasco J‐715 CD spectropolarimeter at 20°C. Each spectrum represents a background subtracted (buffer only) average of four scans. Data were processed with J‐700 Software v1.5 (Jasco) and KaleidaGraph v4.5 (Synergy Software). Percent helicity was calculated from the mean residue ellipticity (MRE) using Equation (1) [47, 48]:

(1) $$\%\text{helicity}=100\times \left(\frac{\mathrm{MRE}222}{\left[-39,500\left(\frac{1-2.57}{n}\right)\right]}\right)$$

where MRE222 is the mean residue ellipticity at 222 nm and *n* is the total number of peptide bonds.

### 2.7 Protein Expression and Purification

Recombinant His‐tagged Bcl‐2 proteins were purified from BL21(DE3) competent cells as described previously [29]. All Bcl‐2 proteins were expressed without their transmembrane domains (ΔTM) to aid expression, purification and solubility [30]. Briefly, cells harboring Bcl‐2‐ΔTM plasmids were grown as 1 L cultures in LB media containing 100 μg/mL ampicillin to an OD_600_ of 0.8 at 37°C. Protein expression was then induced for 4 h at 37°C using 1 mM isopropyl β‐D‐1‐thiogalactopyranoside (IPTG). Following induction, the cells were pelleted and stored at −80°C. To extract the His‐tagged Bcl‐2‐ΔTM proteins, pellets from 500 mL volumes of media were suspended in 10 mL cold bacterial‐protein extraction reagent (B‐PER) supplemented with 10 mM imidazole and protease inhibitor cocktail. This mixture was allowed to shake at 4°C for 10 min before being centrifuged at 15,000 × g for 15 min at 4°C. The cleared lysate was then added across a freshly prepared Ni‐NTA agarose column that had been equilibrated with equilibration buffer (50 mM NaH_2_PO_4_, 500 mM NaCl, 100 mM imidazole, pH 8.0). The suspension was then allowed to incubate for 1 h at 4°C with end‐over‐end rotation. Following incubation, the flow‐through was collected and unbound proteins were removed from the column with 5 column volumes of washing buffer (50 mM NaH_2_PO_4_, 500 mM NaCl, 20 mM imidazole, pH 8). His‐tagged Bcl‐2‐ΔTM proteins were then eluted from the column using 15 mL of elution buffer (50 mM NaH_2_PO_4_, 500 mM NaCl, 250 mM imidazole, pH 8) in 3 mL fractions. Once eluted, the purified proteins were dialyzed into 2 L of binding buffer (50 mM Tris, 100 mM NaCl, pH 8.0). Following dialysis, the Bcl‐2‐ΔTM proteins were concentrated using centrifugal filtration units (Amicon). Final protein concentrations were determined using standard Bradford assays [49]. The overall purity (> 95%) of the proteins was determined by loading 25 μL of the collected fractions onto a 14% polyacrylamide gel and separating the proteins by SDS‐PAGE [29]. Visualization of the proteins was achieved by staining the gel with Coomassie blue (data not shown). Concentrated proteins were aliquoted into fresh microfuge tubes, flash frozen and stored at −80°C until further use.

### 2.8 Competitive Binding Assays

FP competitive binding assays were performed in triplicate on black 384‐well plates (#3575, Corning, Corning, NY) using a SpectraMax M5e multi‐mode plate reader (Molecular Devices, Sunnyvale, CA). For these experiments, 100 nM Bcl‐2‐*Δ*TM and 25 nM ^Flu^Bax‐BH3^ΔB^ were initially co‐incubated for 1 h in binding buffer at ambient temperature in the dark. Following pre‐incubation, serial dilutions of acetylated peptides (100 μM to 2.3 pM) were added, and the solutions were allowed to incubate for an additional 1 h at ambient temperature in the dark. This incubation time was deemed sufficient for binding reactions to reach equilibrium, as judged by an absence of change in the observed polarization value of the sample with the lowest protein concentration over 4 h (data not shown). Following incubation, the fluorescence polarization of each sample was measured using an excitation wavelength of 498 nm and an emission wavelength of 525 nm. An average of 100 reads were recorded for each well. Polarization data were processed using SoftmaxPro v6.4 software (Molecular Devices) and binding curves were fit using Equation (2) [50] in KaleidaGraph v4.5 (Synergy Software):

(2) $${\mathrm{FP}}_{\mathrm{obs}}=\frac{\left(\text{FPmax}-\text{FPmin}\right)}{\left[1+{\left(\frac{{\mathrm{IC}}_{50}}{L}\right)}^{\text{slope}}\right]}+{\mathrm{FP}}_{\min}$$

where *FP*_*obs*_ is the observed fraction of fluorescently‐labeled peptide bound at any competitor peptide concentration, *slope* is the slope at the inflection point, and IC_50_ is the concentration of competitor that reduces binding of fluorescently‐labeled peptide by 50%. *L* is the concentration of peptide, *FP*_*min*_ is minimum FP value, and *FP*_*max*_ is maximum FP value.

### 2.9 Isothermal Titration Calorimetry

All ITC experiments were performed in triplicate using a MicroCal 200 ITC instrument (Malvern Panalytical) at 25°C. To initiate ITC experiments, the sample cell was filled with 250 μL of binding buffer supplemented with 20 μM Bcl‐2‐*Δ*TM. Acetylated peptides were then diluted to a final concentration of 200 μM in binding buffer and injected into the sample cell at 2 μL volumes using a 6 s injection time and a 150 s delay between each injection. Data analysis was performed using MicroCal Origin software v7.0. Non‐linear curves were fit using Equation (3) adapted from the instrument manual:

(3) $$\mathrm{\Delta Q}=\frac{nM_{t}\Delta HV_{o}}{2}\left[1+\frac{X_{t}}{nM_{t}}+\frac{1}{\mathrm{nK}{\mathrm{aM}}_{t}}-\sqrt{{\left(1+\frac{X_{t}}{nM_{t}}+\frac{1}{\mathrm{nKa}M_{t}}\right)}^{2}-\frac{4X_{t}}{nM_{t}}}\right]$$

where, ΔQ is the heat content released or absorbed by each injection. Δ*H* is the enthalpy change of ligand binding and *M*_*t*_ is bulk concentration of Bcl‐2‐ΔTM. *V*_*o*_ is the cell volume of Bcl‐2‐ΔTM and *K*_*a*_ is the affinity constant. *X*_*t*_ is bulk concentration of peptide and *n* is the number of sites occupied by the peptide. Dissociation constants (*K*_d_) for all peptides were calculated by taking the reciprocal of the *K*_*a*_.

Δ*G* values were calculated from the Gibbs free energy Equation (4) using Origin‐fitted values of Δ*H* and program‐calculated values of Δ*S*.

(4) ΔG=ΔΗ−ΤΔS

## 3 Results

### 3.1 Rational Design of ScTx‐Bax Peptides

**FIGURE 1:**
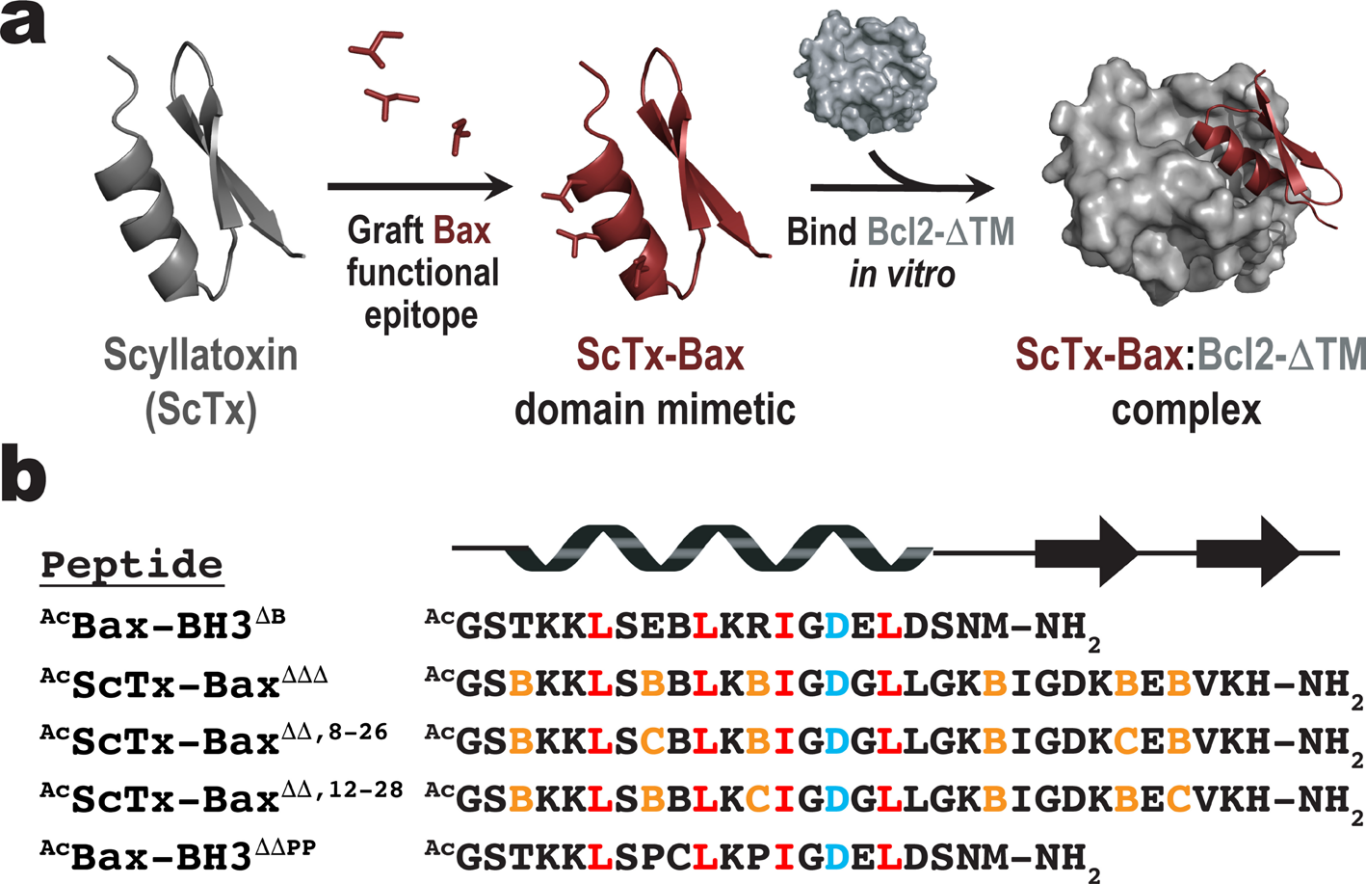
Strategy for targeting Bcl‐2‐ΔTM proteins with ScTx‐Bax BH3 domain mimetics. (a) Bax BH3 residues important for BCL2 recognition are grafted onto the α‐helix of ScTx. The ScTx‐Bax mimetic is then allowed to target the BH3‐binding pocket of Bcl‐2 in vitro. (b) Sequence alignment of Bax BH3 domain peptides and ScTx‐Bax BH3 domain mimetics used in this work. Epitopes required for BCL2 recognition are shown in red; structural cysteines and amino butyric acid residues (B) are orange; conserved BH3 domain aspartic acid is colored cyan.

Our laboratory previously reported the development of ScTx‐Bax BH3 domain mimetics that target Bcl‐2 proteins with low micromolar affinity in vitro [29, 41]. In the current study, we used a similar protein grafting strategy to synthesize three structurally unique ScTx‐Bax peptides for in vitro competitive binding and ITC studies against Bcl‐2. Here, amino acids from the Bax BH3 domain important for Bcl‐2 recognition were aligned with solvent exposed residues within the α‐helix of ScTx. This allowed us to generate an optimized ScTx‐Bax sequence that would target Bcl‐2 within its BH3‐binding pocket (Figure 1a). Importantly, Cys residues required for ScTx folding were aligned with BH3 residues that point away from the BH3:BCL2 interface [18]. This design approach produced ScTx constructs with α‐helices that were near‐perfect sequence mimetics of the Bax BH3 domain (Figure 1b). For the studies outlined herein, we synthesized acetylated (Ac) versions of ScTx‐Bax mimetics that had previously been shown to target Bcl‐2 proteins with relatively high affinity in vitro, including ScTx‐Bax^ΔΔΔ^, ScTx‐Bax^ΔΔ,8–26^ and ScTx‐Bax^ΔΔ,12–28^ [41]. Structurally, ^Ac^ScTx‐Bax^ΔΔΔ^ contains no disulfide linkages, while ^Ac^ScTx‐Bax^ΔΔ,8–26^ and ^Ac^ScTx‐Bax^ΔΔ,12–28^ each include a single disulfide, respectively positioned near the middle (C8—C26) or C‐terminal end (C12—C28) of the ScTx‐Bax α‐helix. Loss of an individual disulfide linkage is indicated by a Δ symbol in the superscript of the peptide nomenclature, while the remaining disulfide linkage (if any) is indicated by the numbers of the cysteine pair. All native ScTx cysteines were replaced with the structural isostere aminobutyric acid (Abu, B) where necessary [42, 51]. As a positive control for our studies, we used the 21‐amino acid peptide ^Ac^Bax‐BH3^ΔB^ that was derived directly from the Bax BH3 domain sequence (residues 55–74). To prevent oxidation of the control peptide, the native C62 residue of ^Ac^Bax‐BH3^ΔB^ was replaced with Abu; this single amino acid change is specified by the ΔB superscript. We also developed ^Ac^Bax‐BH3^ΔΔPP^ as a negative control for Bcl‐2‐ΔTM binding. This peptide includes two prolines (P) that replace native residues E61 and R65 within the Bax BH3 sequence; these two amino acid changes are indicated by the ΔΔPP superscript. Prolines are typically classified as helix disruptors when placed in the primary sequence of structured proteins and peptides [52]. We therefore reasoned that ^Ac^Bax‐BH3^ΔΔPP^ would not bind to Bcl‐2 by virtue of its inability to fold into a helical structure. Finally, we used the previously reported fluorescent peptide ^Flu^Bax‐BH3^ΔB^ [41] as a tracer for FP competitive binding assays.

### 3.2 Synthesis and Folding of ScTx‐Bax Peptides

Once our ScTx‐Bax sequence variants were designed, we used standard Fmoc SPPS procedures to generate the constructs (see Section 2). All peptides used for competitive binding and ITC studies were capped at their N‐terminus with an acetyl group to enhance stability [53]. In order to facilitate disulfide bond formation, reduced isoforms of ^Ac^ScTx‐Bax^ΔΔ,8–26^ and ^Ac^ScTx‐Bax^ΔΔ,12–28^ were subjected to air oxidation in phosphate buffer over 24 h (see Section 2), and the reaction progress was monitored by analytical HPLC (Figures S1a and S1b). Despite requiring oxidation times up to 24 h, each fully‐oxidized ScTx‐Bax variant displayed a well‐defined product peak and showed no indication of multimerization under these conditions. Specifically, we observed that ^Ac^ScTx‐Bax^ΔΔ,8–26^ underwent a moderately robust oxidation, with 83% of the reduced starting material being converted to the final product. On the other hand, nearly 92% of ^Ac^ScTx‐Bax^ΔΔ,12–28^ was oxidized under similar conditions. This result indicates that the C8—C26 disulfide linkage is marginally more difficult to form than the C12—C28 bond, and is likely attributed to comparatively greater flexibility of the C8 and C26 side chains within that region of the ScTx‐Bax backbone [40, 51]. Following completion of the reaction, the oxidized products were identified by MALDI‐MS and purified to > 95% as determined by analytical HPLC chromatograms (Figure S2 and Table S1). The position of the disulfide linkage within oxidized ^Ac^ScTx‐Bax^ΔΔ,8–26^ and ^Ac^ScTx‐Bax^ΔΔ,12–28^ was confirmed by MALDI‐MS analysis of trypsin‐digested fragments as previously described [29] (Figures S3a and S3b). This analysis confirmed that the formation of the single disulfide linkage occurred between residues C8—C26 in ^Ac^ScTx‐Bax^ΔΔ,8–26^ and C12—C28 in ^Ac^ScTx‐Bax^ΔΔ,12–28^. To ensure minimal background of contaminating peptide fragments and to mitigate non‐specific cleavage of our target peptides [54], an analogous protease solution without peptide was similarly analyzed as a negative control. This solution showed no autolytic trypsin fragments following 2 h incubation at 37°C, thus confirming the integrity of our experimental samples (data not shown).

### 3.3 Structural Analysis of ScTx‐Bax Peptides

**FIGURE 2:**
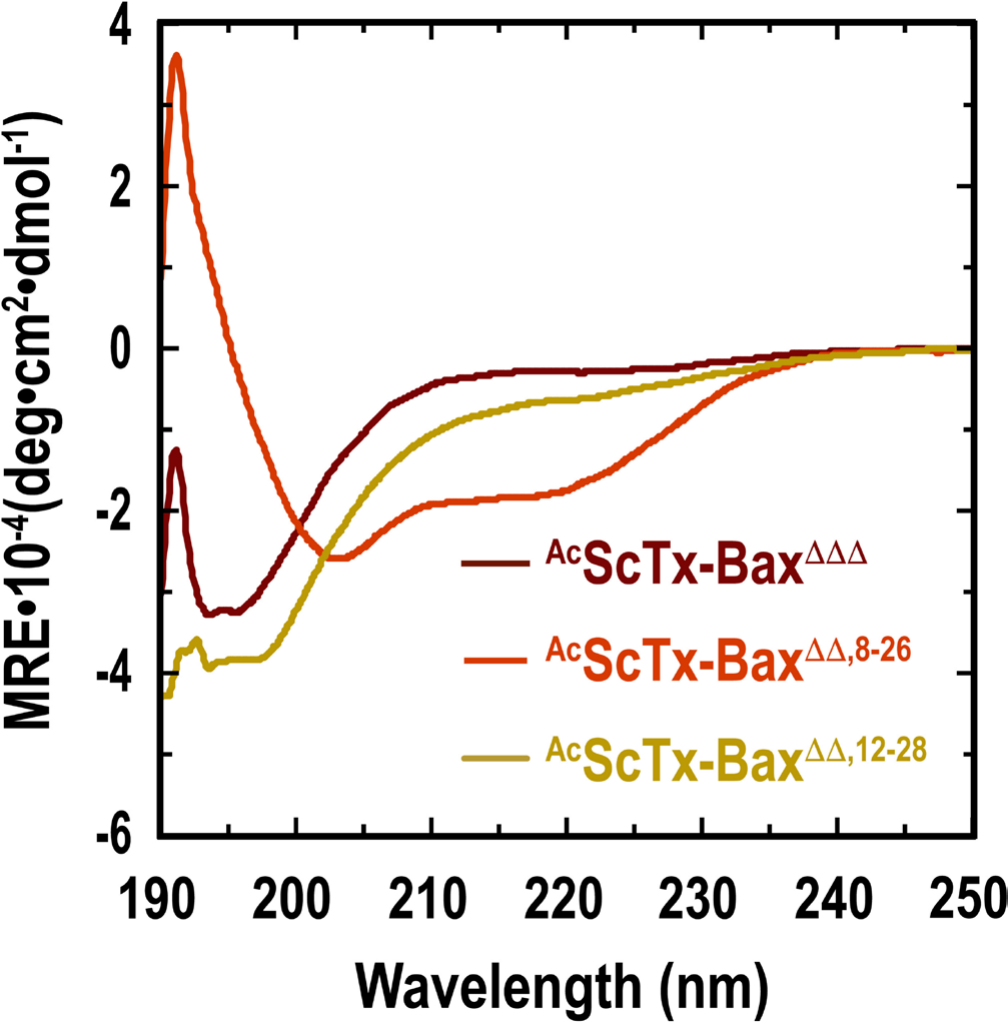
Wavelength‐dependent CD spectra of acetylated ScTx‐Bax proteins (10 μM) in binding buffer (50 mM Tris, 0.1 M NaCl, pH 8). All spectra were acquired at 20°C and represent an average of four background‐subtracted (buffer only) scans.

We next evaluated the structures of our acetylated ScTx‐Bax peptides in solution using wavelength‐dependent CD spectropolarimetry. These studies were performed by dissolving each peptide in binding buffer to a final concentration of 10 μM and measuring the mean‐residue ellipticity (MRE) at 20°C in the far‐UV range (190–250 nm). Control peptides ^Ac^Bax‐BH3^ΔB^ and ^Ac^Bax‐BH3^ΔΔPP^ each displayed spectra that were indicative of random coil structures under these conditions, with a negative minimum at 195 nm and a shoulder at 220 nm (Figure S4a). We next evaluated the helical propensity of the control peptides by incubating them in binding buffer supplemented with 30% TFE. Here, the CD spectrum of ^Ac^Bax‐BH3^ΔB^ showed a positive maximum at 192 nm and negative double minima at 204 nm and 216 nm, indicating that this peptide is capable of transitioning to an α‐helix in the presence of structure inducing co‐solvents (Figure S4b). On the contrary, ^Ac^Bax‐BH3^ΔΔPP^ was not able to fold into an α‐helix even in the presence of 30% TFE and displayed a CD signature comparable to that observed in binding buffer alone (Figure S4b). The structures of ^Ac^ScTx‐Bax^ΔΔΔ^, ^Ac^ScTx‐Bax^ΔΔ,8–26^ and ^Ac^ScTx‐Bax^ΔΔ,12–28^ were similarly evaluated using wavelength‐dependent CD spectropolarimetry (Figure 2). Here, it was observed that ^Ac^ScTx‐Bax^ΔΔΔ^ displayed a CD signature suggestive of a random coil structure, with a negative minimum at 195 nm and a slight shoulder at 225 nm. ^Ac^ScTx‐Bax^ΔΔ,12–28^, which contains a single disulfide near the C‐terminus of its α‐helix also displayed a CD spectrum that was indicative of a random coil, with a negative minimum at 196 nm and a slight shoulder at 222 nm. Notably, ^Ac^ScTx‐Bax^ΔΔ,8–26^ displayed a CD spectrum that was reminiscent of those obtained for fully‐oxidized wild‐type ScTx proteins [29, 51], with a positive maximum at 191 nm, a negative minimum at 203 nm, and a pronounced shoulder at 220 nm. This result supports our previous findings that a single disulfide linkage located at position C8—C26 rescues the α/β structural motif observed with fully‐oxidized wild‐type ScTx peptides. These findings also suggest that isolated ^Ac^ScTx‐Bax^ΔΔ,8–26^ peptides present the grafted Bax BH3 domain as a well‐ordered helix. Finally, the percent helicity of our ScTx‐Bax peptides were calculated from the observed CD spectra using Equation (1) [47, 48] (see Section 2). Here, we found that ^Ac^ScTx‐Bax^ΔΔΔ^ was 13.1% helical under these conditions, while ^Ac^ScTx‐Bax^ΔΔ,8–26^ and ^Ac^ScTx‐Bax^ΔΔ,12–28^ were found to be 39.3% and 14.2% helical, respectively. These results indicate that installing a disulfide linkage at position C8—C26 within the primary sequence forces a significant percentage of ^Ac^ScTx‐Bax^ΔΔ,8–26^ peptides to fold into an α/β structural motif. Furthermore, these results suggest that despite being predominantly disordered in solution, a slightly higher percentage of ^Ac^ScTx‐Bax^ΔΔ,12–28^ molecules adopt helical folds compared to ^Ac^ScTx‐Bax^ΔΔΔ^ variants.

### 3.4 ScTx‐Bax Peptides Compete With Bax BH3 Domains for Bcl‐2 Proteins

**FIGURE 3:**
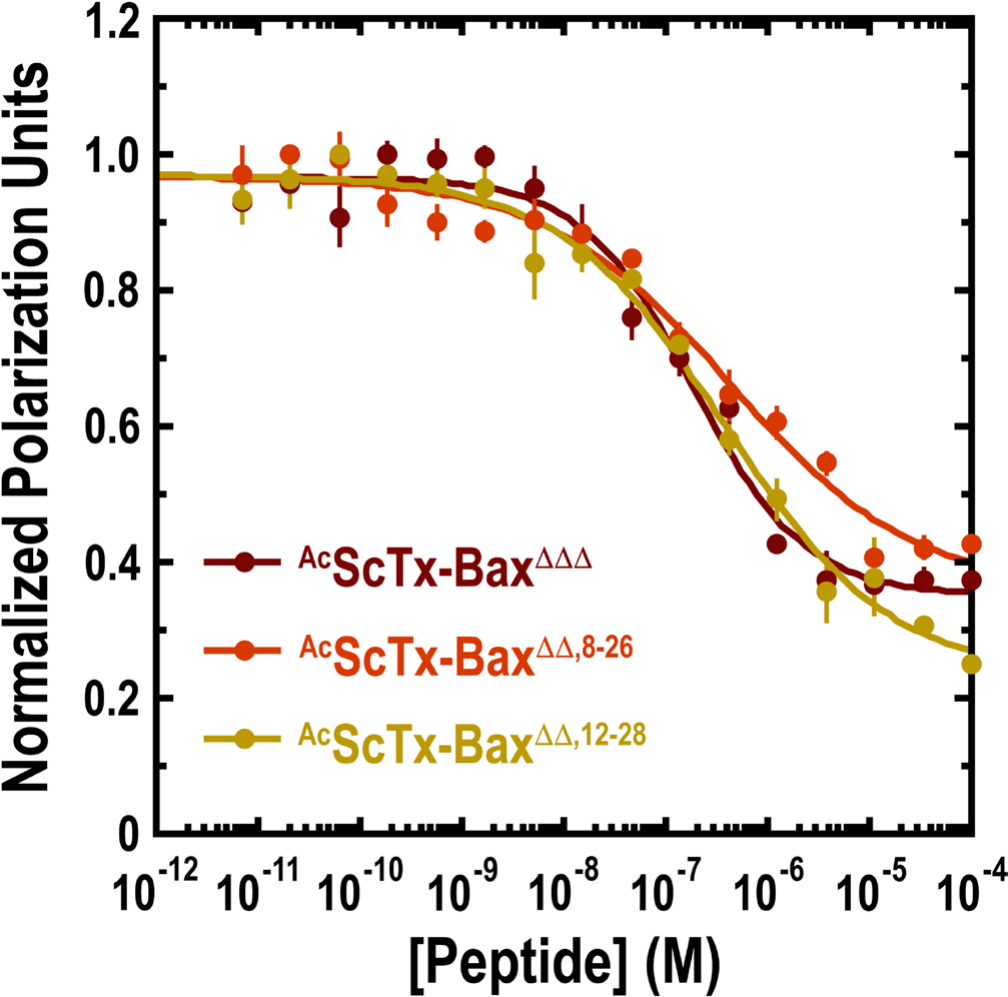
Acetylated ScTx‐Bax peptides compete for the BH3‐binding pocket of Bcl‐2‐ΔTM. Competitive binding of ScTx‐Bax variants to Bcl‐2‐ΔTM was determined using 100 nM protein and 25 nM ^Flu^Bax‐BH3^ΔB^ in binding buffer (50 mM Tris, 0.1 M NaCl, pH 8). Data points represent an average of three independent experiments; error bars are standard deviation.

The ability for our ScTx‐Bax constructs to compete with Bax BH3 domain peptides for the BH3‐binding pocket of Bcl‐2‐ΔTM was assessed in vitro using FP competitive binding assays. Under these conditions, the acetylated control peptide ^Ac^Bax‐BH3^ΔB^ was found to compete with fluorescently‐labeled ^Flu^Bax‐BH3^ΔB^ for Bcl‐2‐ΔTM with an IC_50_ of 141.0 nM (Table 1, Figure S5). This IC_50_ value indicates that the ^Ac^Bax‐BH3^ΔB^ construct interacts favorably with Bcl‐2‐ΔTM and is capable of targeting the BH3 binding pocket at moderately low concentrations. As expected, the negative control peptide ^Ac^Bax‐BH3^ΔΔPP^ did not compete with ^Flu^Bax‐BH3^ΔB^ for Bcl‐2‐ΔTM under these conditions and therefore no IC_50_ value was determined for this construct (Table 1, Figure S5). All ScTx‐Bax peptides tested were found to compete with ^Flu^Bax‐BH3^ΔB^ for Bcl‐2‐ΔTM; however, each variant inhibited the interaction with varying degrees of efficacy (Figure 3). For example, ^Ac^ScTx‐Bax^ΔΔΔ^ displayed an IC_50_ value of 184.6 nM, while ^Ac^ScTx‐Bax^ΔΔ,8–26^ and ^Ac^ScTx‐Bax^ΔΔ,12–28^ showed respective IC_50_ values of 427.5 and 377.4 nM (Table 1). It is notable that the unstructured ^Ac^ScTx‐Bax^ΔΔΔ^ peptide targets Bcl‐2‐ΔTM with affinity similar to that of our control peptide ^Ac^Bax‐BH3^ΔB^, despite including only the first 16 amino acids (44%) of the full‐length helical Bax BH3 domain [18]. In contrast, the more constrained BH3 domain mimetics ^Ac^ScTx‐Bax^ΔΔ,8–26^ and ^Ac^ScTx‐Bax^ΔΔ,12–28^ suffered modest losses in inhibitory potency, with the unstructured (albeit constrained) ^Ac^ScTx‐Bax^ΔΔ,12–28^ variant displaying a lower IC_50_ value than the fully‐structured ^Ac^ScTx‐Bax^ΔΔ,8–26^. These findings are in good agreement with our previous direct binding studies [29, 41] in which unstructured ScTx‐Bax variants targeted Bcl‐2‐ΔTM with the highest affinity. Taken together, these data suggest that ^Ac^ScTx‐Bax^ΔΔ,8–26^ variants fold into more rigid structures than those with no disulfides or disulfides positioned near the C‐terminus of the ScTx α‐helix. It also follows from these observations that favorable BH3:BCL2 interactions occur through an induced‐fit binding mechanism, as locking the N‐terminal region of the Bax BH3 domain (residues 54–70) into a rigidified structure is detrimental to achieving optimal association to Bcl‐2 proteins.

**TABLE 1:** Competitive binding and thermodynamic parameters for acetylated BH3 domain peptides targeting Bcl‐2‐ΔTM in vitro.

Peptide	IC_50_ (nM)^a^	*n*	Δ*G* (kcal/mol)	Δ*H* (kcal/mol)	TΔ*S* (kcal/mol)	*K*_d_ (nM)^b^
^Ac^Bax‐BH3^ΔB^	141.0	0.93 ± 0.05	−10.02 ± 0.89	−5.43 ± 0.73	4.58 ± 0.20	191.2 ± 27.5
^Ac^ScTx‐Bax^ΔΔΔ^	184.6	0.90 ± 0.09	−8.49 ± 0.33	−3.85 ± 0.33	4.64 ± 0.11	296.5 ± 22.9
^Ac^ScTx‐Bax^ΔΔ,8–26^	427.5	1.13 ± 0.08	−7.51 ± 0.12	−5.88 ± 0.15	1.63 ± 0.03	3428.4 ± 256.9
^Ac^ScTx‐Bax^ΔΔ,12–28^	377.4	1.20 ± 0.02	−8.38 ± 0.33	−5.57 ± 0.45	2.81 ± 0.11	502.2 ± 30.3
^Ac^Bax‐BH3^ΔΔPP^	N.B.	N.B	N.B.	N.B.	N.B.	N.B

*Note: n*, number of sites; N.B. indicates no binding; ± are standard deviation (number of trials = 3).

^a^ Inhibitory concentrations (IC50) were determined from competitive binding assays.

^b^ Dissociation constants (*K*_d_) were calculated from ITC experiments by taking the reciprocal of the affinity constant (*K*_*a*_).

### 3.5 Binding Thermodynamics of ScTx‐Bax Peptides Targeting Bcl‐2 Proteins

**FIGURE 4:**
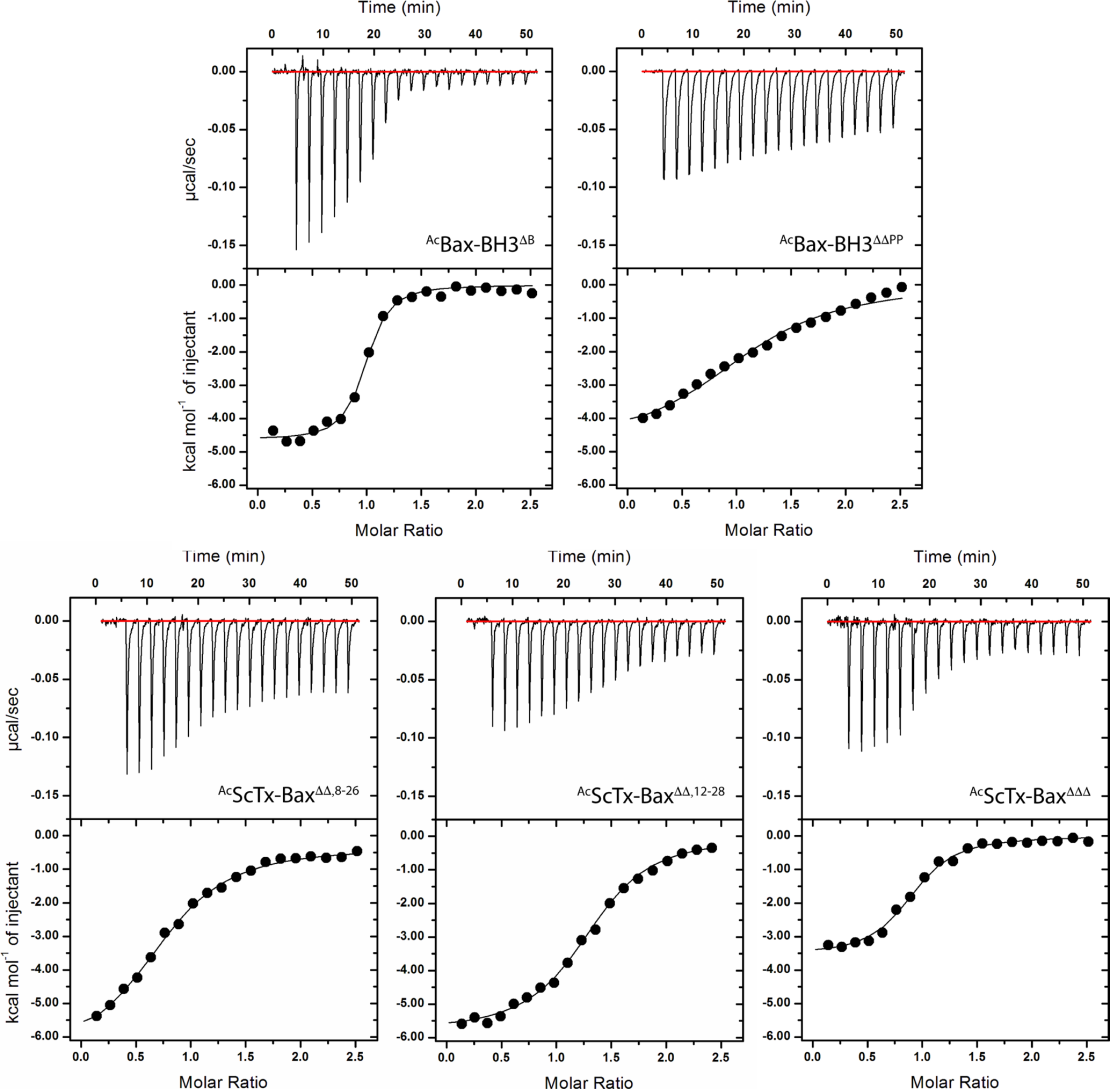
Representative isotherms for the binding of BH3 domain peptides to the repressor protein Bcl‐2‐ΔTM at 25°C. Upper panels show raw ITC data expressing change in thermal power as a function of time. Lower panels display change in molar heat expressed as a function of molar ratio (peptide to Bcl‐2‐ΔTM). Solid lines in the lower panels show non‐linear least squares fit of data to a one‐site binding model using Equation (3) (see Section 2).

Given that each ScTx‐Bax mimetic possesses varying degrees of structural rigidity, we reasoned that these constructs could be used to assess how flexibility within the Bax BH3 domain affects the thermodynamics of binding to Bcl‐2‐ΔTM. To this end, we employed ITC to gain insight into the binding thermodynamics of ScTx‐Bax mimetics that target Bcl‐2‐ΔTM in vitro. All ITC experiments were performed in triplicate at 25°C in binding buffer (see Section 2). Briefly, acetylated peptides were titrated periodically into binding buffer supplemented with Bcl‐2‐ΔTM, and binding isotherms were generated by monitoring the heat change of the system as a function of time (Figure 4). All acetylated ScTx‐Bax peptides and the control ^Ac^Bax‐BH3^ΔB^ peptide displayed sigmoidal binding isotherms when mixed with Bcl‐2‐ΔTM. Not surprisingly, the unstructured peptide ^Ac^Bax‐BH3^ΔΔPP^ displayed a fairly linear isotherm that remained unsaturated even at higher concentrations, indicating that this peptide does not interact favorably with Bcl‐2‐ΔTM under these conditions. Thermodynamic parameters such as molar ratios (*n*), affinity constants (*K*_a_), changes in Gibbs free energy (Δ*G*), enthalpy (Δ*H*) and entropy (TΔ*S*) were calculated from non‐linear regression curve‐fitting of the binding isotherms (Table 1, Figure 4).

Our analysis of the thermodynamic data was initiated by determining *n* and dissociation constant (*K*_d_) values for each peptide construct. All *K*_d_ values were determined by taking the reciprocal of the *K*_a_ [55]. Data extracted from the binding isotherms in Figure 4 indicated that all peptides tested (with the exception of ^Ac^Bax‐BH3^ΔΔPP^) bound Bcl‐2‐ΔTM with *n* values ranging from 0.93 ± 0.05 to 1.20 ± 0.02 (Table 1). These results, combined with our competitive binding data (Figure 3, Table 1), strongly suggest that each peptide targets the BH3‐binding groove of Bcl‐2‐ΔTM at a 1:1 molar ratio. It was also shown that our peptide constructs had relatively high affinity for Bcl‐2‐ΔTM, with *K*_d_ values ranging from 191.2 ± 27.5 nM to 3428.4 ± 256.9 nM (Table 1). Specifically, ^Ac^Bax‐BH3^ΔB^ had the highest affinity for Bcl‐2‐ΔTM among all peptides tested at 191.2 ± 27.5 nM, which indicates that the native BH3 domain of Bax is able to target Bcl‐2‐ΔTM with greater efficacy than any of the ScTx‐Bax mimetics tested under these conditions. On the other hand, the unstructured ^Ac^ScTx‐Bax^ΔΔΔ^ peptide was observed to bind Bcl‐2‐ΔTM with only slightly lower affinity at 296.5 ± 22.9 nM, but still had the highest affinity for Bcl‐2‐ΔTM among any of the ScTx‐Bax variants tested. Each of the oxidized ScTx‐Bax peptides showed modest binding affinity for Bcl‐2‐ΔTM, with ^Ac^ScTx‐Bax^ΔΔ,8–26^ and ^Ac^ScTx‐Bax^ΔΔ,12–28^ targeting the protein at 3428.4 ± 256.9 nM and 502.2 ± 30.3 nM respectively. Notably, this trend in binding affinity among structured and unstructured ScTx‐Bax BH3 domain mimetics was similarly observed previously using direct FP binding experiments [41], and further supports the notion that favorable BH3:BCL2 interactions are facilitated when the BH3 domains remain flexible [22, 29].

**FIGURE 5:**
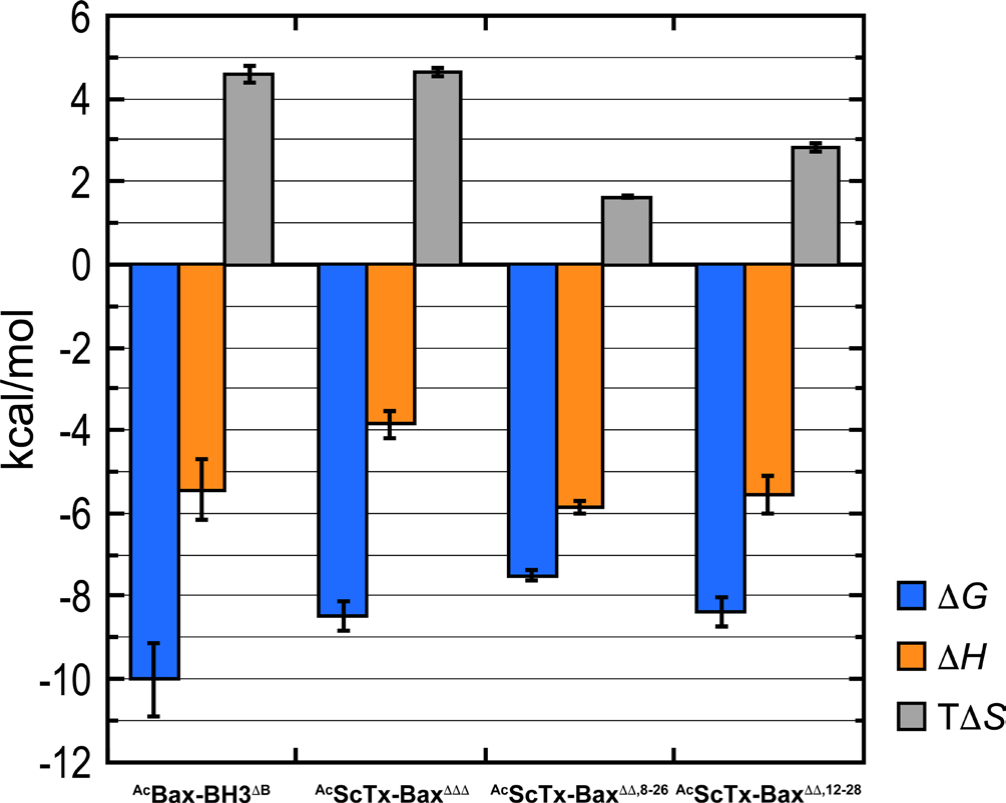
Enthalpic and entropic contributions to the Gibbs free energy of acetylated BH3 domain mimetics binding to Bcl‐2‐ΔTM. The blue, orange and grey bars represent the respective changes in Gibbs free energy (Δ*G*), enthalpy (Δ*H*) and entropy (TΔ*S*) of binding at 25°C; error bars are standard deviation.

Each peptide tested herein showed favorable thermodynamics of binding to Bcl‐2‐ΔTM, with negative Δ*G* values, negative Δ*H* values, and positive Δ*S* values; (Table 1, Figure 5). Furthermore, all errors reported for our thermodynamic parameters were within range of similar ITC experiments reported previously [56, 57]. At this point, we recognized that effectively comparing thermodynamic parameters such as Δ*G*, Δ*H* and Δ*S* among ligands with different physicochemical characteristics is a considerable challenge [58]. For example, the dynamic structural properties between ^Ac^Bax‐BH3^ΔB^ and ^Ac^ScTx‐Bax^ΔΔ,8–26^ are so varied that it would be especially difficult to interpret a structure‐based comparison of their respective thermodynamic binding data. Therefore, we limit our analysis herein to comparing the thermodynamic properties of the two fully‐unstructured peptides (^Ac^Bax‐BH3^ΔB^ and ^Ac^ScTx‐Bax^ΔΔΔ^) and the three ScTx‐Bax peptides (^Ac^ScTx‐Bax^ΔΔΔ^, ^Ac^ScTx‐Bax^ΔΔ,8–26^ and ^Ac^ScTx‐Bax^ΔΔ,12–28^) separately. Using this approach, we observed that the two unstructured peptides, ^Ac^Bax‐BH3^ΔB^ and ^Ac^ScTx‐Bax^ΔΔΔ^, targeted Bcl‐2‐ΔTM with respective Δ*G* values of −10.02 ± 0.89 kcal/mol and −8.49 ± 0.33 kcal/mol. These results suggest that both unstructured peptides interact spontaneously with Bcl‐2‐ΔTM, however, the ^Ac^Bax‐BH3^ΔB^ complex was more stable by 1.53 kcal/mol. Additional thermodynamic parameters obtained from our ITC experiments indicated that the interactions between Bcl‐2‐ΔTM and the fully‐unstructured peptides are driven by favorable changes in both enthalpy and entropy (Table 1, Figure 5). It has been suggested that favorable changes in enthalpy are associated with higher incidences of electrostatic and van der Waals interactions upon protein association [58, 59]. Alternatively, the entropic contributions to molecular interactions are thought to be primarily influenced by solvation entropy gain and ligand entropy loss [60, 61]. These respective entropic parameters reflect changes in the desolvation status at the binding interface and conformation of the molecules upon association. Specifically, binding of ^Ac^Bax‐BH3^ΔB^ to Bcl‐2‐ΔTM resulted in a Δ*H* of −5.43 ± 0.73 kcal/mol and a TΔ*S* of 4.58 ± 0.20 kcal/mol. The relatively large negative enthalpy change observed for this interaction suggests that ^Ac^Bax‐BH3^ΔB^ binds strongly with Bcl‐2‐ΔTM, and that the association is driven primarily by electrostatic and van der Waals interactions. It should also be noted that a gain or loss in net entropy is typically determined by the balance between the gain in solvation entropy and the losses in conformational, translational and rotational entropies of the binding partners. Moreover, favorable changes in entropy upon ligand binding are associated with desolvation of hydrophobic interactions and desirable conformational changes [23]. The favorable entropic value observed when ^Ac^Bax‐BH3^ΔB^ binds to Bcl‐2‐ΔTM therefore indicates a greater gain in solvation entropy and suggests that the entropy gained from ejecting water molecules from the interface surface is greater than the entropy lost from rigidifying the peptide upon association [23]. Analysis of the ^Ac^ScTx‐Bax^ΔΔΔ^ isotherm showed that binding to Bcl‐2‐ΔTM resulted in favorable Δ*H* and TΔ*S* values of −3.85 ± 0.33 kcal/mol and 4.64 ± 0.11 kcal/mol, respectively. These results indicate that association between ^Ac^ScTx‐Bax^ΔΔΔ^ and Bcl‐2‐ΔTM proteins is driven primarily by changes in ligand conformation and desolvation of hydrophobic interactions at the binding interface. Interestingly, the enthalpic contribution for ^Ac^ScTx‐Bax^ΔΔΔ^ binding to Bcl‐2‐ΔTM was 1.58 kcal/mol less favorable compared to that observed with ^Ac^Bax‐BH3^ΔB^, while the entropic contributions observed for these two molecules were effectively equal. This indicates that the comparatively more favorable Δ*G* value observed for the ^Ac^Bax‐BH3^ΔB^ peptide is driven primarily by more favorable enthalpic contributions to the interaction. Finally, if we assume comparable losses in translational and rotational entropies from the two peptides, the more favorable enthalpy value for ^Ac^Bax‐BH3^ΔB^ suggests a greater burial of surface area upon binding, leading to a larger solvation entropy gain. As a consequence, the comparable net entropy change for both peptides indicates that ^Ac^Bax‐BH3^ΔB^ binding to Bcl‐2‐ΔTM likely involves a greater loss in conformational entropy compared to ^Ac^ScTx‐Bax^ΔΔΔ^.

We next turned our attention to comparing the thermodynamic profiles of our three ScTx‐Bax structural variants. It has been shown previously that structurally‐constrained ligands can experience reduced entropic penalties upon binding to cognate proteins compared to their unstructured counterparts [62, 63, 64]. Therefore, our initial hypothesis was that the loss of entropy upon binding the structured ^Ac^ScTx‐Bax^ΔΔ,8–26^ variant to Bcl‐2‐ΔTM would be lower compared to the unstructured ^Ac^ScTx‐Bax^ΔΔ,12–28^ and ^Ac^ScTx‐Bax^ΔΔΔ^ peptides. An analysis of the binding thermodynamics between our ScTx‐Bax mimetics revealed that ^Ac^ScTx‐Bax^ΔΔΔ^ bound Bcl‐2‐ΔTM with a Δ*G* of −8.49 ± 0.33 kcal/mol, while the Δ*G* values for ^Ac^ScTx‐Bax^ΔΔ,8–26^ and ^Ac^ScTx‐Bax^ΔΔ,12–28^ were −7.51 ± 0.12 kcal/mol and −8.38 ± 0.33 kcal/mol, respectively. Interestingly, the Gibbs free energy of binding between the fully unstructured ^Ac^ScTx‐Bax^ΔΔΔ^ and Bcl‐2‐ΔTM was nearly identical to that observed for the ^Ac^ScTx‐Bax^ΔΔ,12–28^ variant, which contains a single disulfide linkage. In addition, these data also revealed that ^Ac^ScTx‐Bax^ΔΔΔ^ binds Bcl‐2‐ΔTM more favorably than the fully‐structured ^Ac^ScTx‐Bax^ΔΔ,8–26^ variant by nearly 1 kcal/mol. Despite the fact that the Gibbs free energy of binding was found to be largely similar among all three ScTx‐Bax variants, our results show that the ^Ac^ScTx‐Bax^ΔΔΔ^ peptide binds to Bcl‐2‐ΔTM more favorably than its disulfide‐containing counterparts. We also observed disparate enthalpic contributions to binding for each construct (Table 1, Figure 5). Specifically, the oxidized ^Ac^ScTx‐Bax^ΔΔ,8–26^ and ^Ac^ScTx‐Bax^ΔΔ,12–28^ variants each had similar Δ*H* values of −5.88 ± 0.15 kcal/mol and − 5.57 ± 0.45 kcal/mol, respectively. Alternatively, the unstructured ^Ac^ScTx‐Bax^ΔΔΔ^ variant displayed the least negative, and therefore least favorable, Δ*H* value at −3.85 ± 0.33 kcal/mol. The binding of each ScTx‐Bax peptide to Bcl‐2‐ΔTM was also characterized by favorable positive TΔ*S* values (Table 1, Figure 5), indicating that the binding to Bcl‐2‐ΔTM was entropically favorable for each construct. Nevertheless, the values for TΔ*S* varied considerably among the three peptides tested, ranging from 1.63 ± 0.03 kcal/mol to 4.64 ± 0.11 kcal/mol. To our surprise, we observed that the constrained peptide ^Ac^ScTx‐Bax^ΔΔ,8–26^ had the least favorable entropic contribution to binding at 1.63 ± 0.03 kcal/mol, while the constrained but largely unstructured ^Ac^ScTx‐Bax^ΔΔ,12–28^ variant showed a more favorable entropic value at 2.81 ± 0.11 kcal/mol. Moreover, we found that the fully unstructured variant ^Ac^ScTx‐Bax^ΔΔΔ^ had the most favorable entropy of binding at 4.64 ± 0.11 kcal/mol, indicating that the unstructured variant experiences a markedly lower entropic penalty when binding Bcl‐2‐ΔTM compared to its oxidized counterparts. Collectively, these results suggest that the forces that accompany favorable enthalpic changes, such as electrostatic and van der Waals interactions, play a significant role in the interaction between fully structured ScTx‐Bax peptides and Bcl‐2‐ΔTM. Indeed, targeting proteins with ligands that have more rigid structures has been associated with favorable enthalpic changes within the system [65, 66]. We therefore surmise that the pre‐organized structure of ^Ac^ScTx‐Bax^ΔΔ,8–26^ allows for comparatively more enthalpy‐driven interactions with Bcl‐2‐ΔTM than are observed with unstructured ^Ac^ScTx‐Bax variants. Contrarily, interactions between the unstructured ^Ac^ScTx‐Bax^ΔΔΔ^ peptide and Bcl‐2‐ΔTM are accompanied by more favorable changes in entropy within the system.

## 4 Discussion

In this work, a series of ScTx‐Bax sequence variants were developed and used to assess how structural flexibility within the helical Bax BH3 domain affects BH3:BCL2 interactions. Analysis by CD spectropolarimetry showed that the helical propensity of our ScTx‐Bax mimetics is heavily influenced by the number and position of disulfide linkages included within the primary sequence of the peptide. For example, ^Ac^ScTx‐Bax^ΔΔΔ^ peptides contain no disulfide bonds and were observed to adopt random coils in solution. Moreover, the ^Ac^ScTx‐Bax^ΔΔ,12–28^ variant includes a disulfide bond near the C‐terminus of the ScTx α‐helix; however, we found that placing the linkage at this position does not cause a significant percentage of the molecules to adopt an organized structure. On the other hand, it was observed that ^Ac^ScTx‐Bax^ΔΔ,8–26^ folds into an α/β structural motif that is similar to that of fully‐oxidized wild‐type ScTx proteins [29, 51]. This result further supports our previous findings that the C8—C26 disulfide linkage is highly influential for facilitating the folding of ScTx‐based Bax BH3 domain mimetics into native structures [41].

Competitive binding assays showed that all three ScTx‐Bax constructs developed herein compete with isolated Bax BH3 domain peptides for the BH3‐binding pocket of Bcl‐2‐ΔTM in vitro. Specifically, it was observed that the unstructured ^Ac^ScTx‐Bax^ΔΔΔ^ variant inhibited the Bax‐BH3:Bcl‐2 interaction with an IC_50_ of 184.6 nM, which was the lowest value observed for any ScTx‐Bax peptide tested. ScTx‐Bax mimetics with single disulfide linkages displayed IC_50_ values that were up to 2.3‐fold higher than that obtained with the fully unstructured variant. For example, ^Ac^ScTx‐Bax^ΔΔ,12–28^ did not display any ordered folding in solution, but had a comparatively higher IC_50_ value (377.5 nM) than ^Ac^ScTx‐Bax^ΔΔΔ^, which indicates that placing a covalent bond in this region of the ScTx‐Bax structure negatively influences its association with Bcl‐2 proteins in vitro. Surprisingly, ^Ac^ScTx‐Bax^ΔΔ,8–26^, which is presumed to fold into a stable α/β structural motif and likely presents the Bax BH3 domain as an ordered helix, had the highest IC_50_ value (427.5 nM) of all the peptides tested. Taken together, these results indicate that forcing the ScTx‐Bax peptide into a rigid structure adversely affects Bcl‐2‐ΔTM recognition and that an induced‐fit binding mechanism is, at least in part, required for favorable BH3:BCL2 interactions.

We next performed ITC to evaluate the thermodynamics of binding between our ScTx‐Bax peptides and Bcl‐2‐ΔTM in vitro. Results from our ITC experiments indicated that all peptides, with the exception of the negative control peptide ^Ac^Bax‐BH3^ΔΔPP^, bound Bcl‐2‐ΔTM at a 1:1 molar ratio with *K*_d_ values in the low micromolar range. Additional results from ITC experiments revealed that all peptides tested displayed favorable (negative) changes in the Gibbs free energy of binding to Bcl‐2‐ΔTM, with the most energetically favorable binding being observed with the unstructured peptides ^Ac^Bax‐BH3^ΔB^ and ^Ac^ScTx‐Bax^ΔΔΔ^. Despite observing relatively similar Δ*G* values among ^Ac^Bax‐BH3^ΔB^ and ^Ac^ScTx‐Bax^ΔΔΔ^, there were differences in the enthalpic and entropic contributions to binding between these two peptides. More specifically, the enthalpy of binding for ^Ac^Bax‐BH3^ΔB^ to Bcl‐2‐ΔTM was more favorable compared to ^Ac^ScTx‐Bax^ΔΔΔ^, indicating that the changes in electrostatic and van der Waals interactions were more significant upon association of the native BH3 domain peptide. Molecular modeling alignments show that the BH3 domain segment of our ScTx‐Bax peptides only interact with roughly 50% of the BH3 binding groove of Bcl‐2‐ΔTM (Figure 1a). Furthermore, our ScTx‐Bax constructs mimic only the first 16 residues (44%) of the full‐length Bax BH3 domain. The moderately large difference in Δ*H* is therefore presumably related to ^Ac^Bax‐BH3^ΔB^ interacting across a larger surface area within the BH3‐binding pocket compared to ^Ac^ScTx‐Bax^ΔΔΔ^ (Figure 1a). Indeed, this binding configuration likely results in the formation of more electrostatic and van der Waals interactions along the entire BH3:BCL2 interface. Notably, the entropic contribution to ^Ac^Bax‐BH3^ΔB^ interacting with Bcl‐2‐ΔTM is nearly identical to that observed with ^Ac^ScTx‐Bax^ΔΔΔ^, which indicates that the combined effects of desolvation and conformational changes are similarly influential to the binding thermodynamics of ^Ac^Bax‐BH3^ΔB^ and ^Ac^ScTx‐Bax^ΔΔΔ^.

Upon assessing the binding thermodynamics of our ScTx‐Bax structural variants, we discovered that all three constructs showed favorable interaction energies when targeting Bcl‐2‐ΔTM in vitro. However, it was observed that the Gibbs free energy of binding for the fully‐structured ^Ac^ScTx‐Bax^ΔΔ,8–26^ peptide was less favorable compared to ^Ac^ScTx‐Bax^ΔΔ,12–28^ and ^Ac^ScTx‐Bax^ΔΔΔ^. Therefore, the thermodynamic profiles obtained from these experiments suggest that rigidifying the N‐terminal segment of helical BH3 domains negatively impacts its association with Bcl‐2‐ΔTM. Interestingly, the enthalpic contribution to the interaction was found to be most favorable with ^Ac^ScTx‐Bax^ΔΔ,8–26^, and least favorable with the ^Ac^ScTx‐Bax^ΔΔΔ^ peptide. This finding indicates that the structured ScTx‐Bax variant can form more favorable electrostatic and van der Waals interactions with Bcl‐2‐ΔTM proteins compared to its unstructured counterpart. Moreover, this result suggests that enthalpic contributions to BH3:BCL2 interactions can be improved by rigidifying the structure of the ligand when targeting this specific region of the BH3 binding groove. Although the enthalpic contribution (or enthalpic loss) is the most favorable among all peptides tested, ^Ac^ScTxBax^ΔΔ,8–26^ shows the smallest entropy gain, which is likely due to a large conformational entropy loss from rigidification. Consequently, the reduced entropy gain results in a less favorable binding free energy compared to the other peptides, despite its highly favorable enthalpic contribution. Finally, the entropic contribution to binding was most favorable for the fully unstructured ^Ac^ScTx‐ Bax^ΔΔΔ^ and was less favorable for ^Ac^ScTx‐Bax^ΔΔ,8–26^ and ^Ac^ScTx‐Bax^ΔΔ,12–28^. This trend suggests that conformational entropy changes and desolvation effects are more influential to the binding of ScTx‐Bax mimetics that lack disulfide linkages, which otherwise impose structural rigidity.

In summary, this study has provided new insights into the molecular nature of BH3:BCL2 recognition and further expands the utility of ScTx‐based BH3 domain mimetics as tools to study therapeutically relevant protein–protein interactions. Indeed, our analysis of ScTx‐Bax structural variants suggests that the molecular recognition elements of such constructs can be tuned to enhance inhibitory potential and binding thermodynamics when targeting repressor BCL2 proteins in vitro. Specifically, it was determined that rigidifying the N‐terminal segment of the Bax BH3 domain negatively impacted binding to Bcl‐2, despite the fully‐structured variants holding a modest enthalpic advantage over the unstructured constructs. Furthermore, we found that the unstructured (and presumably more flexible) ScTx‐Bax constructs bound Bcl‐2 with greater efficacy. Taken together, these results support the notion that disorder‐to‐order transitions upon binding are required for favorable BH3:BCL2 interactions and that coupled folding/binding is prevalent among BCL2 members [22, 29, 67].

Finally, we expect that ScTx‐Bax BH3 domain mimetics will be uniquely suited for targeting BH3:BCL2 interactions in live cells or in vivo. For instance, ^Ac^ScTx‐Bax^ΔΔ,8–26^ peptides are likely to be more proteolytically stable and cell permeable than its unstructured counterparts owing to its compact, folded architecture ([68, 69]). However, the single disulfide bond of ^Ac^ScTx‐Bax^ΔΔ,8–26^ has the potential to be reduced inside cells [70], turning the originally structured (and less efficacious binder) into an inherently disordered BH3 domain mimetic that has high binding affinity. Moreover, we anticipate that other physicochemical properties, such as polarity and hydrophobicity, can be further modified in ScTx‐Bax mimetics to reduce the promiscuous nature of effector BH3 ligands. To achieve this goal, studies in our laboratory are currently focused on developing highly specific ScTx‐based BH3 domain mimetics that will not only target discrete repressor BCL2 paralogs, but also specific sub‐regions within their respective BH3‐binding pockets.

## Author Contributions

H.A.D.B.A., D.A., M.J.K.V. and J.M.H. designed the research, analyzed the data, and wrote the manuscript. J.M.H directed the design and development of the ScTx‐Bax BH3 domain mimetics. H.A.D.B.A., M.J.K.V. and D.A. synthesized the peptides and characterized the constructs. H.A.D.B.A., M.J.K.V. and D.A. conducted the in vitro expression, purification, and structural studies of the Bcl‐2‐ΔTM proteins. H.A.D.B.A. and D.A. performed all in vitro assays, including circular dichroism, competitive binding and isothermal titration calorimetry. All contributing authors edited the manuscript and approved its final version before submission.

## Ethics Statement

The authors have nothing to report.

## Consent

The authors have nothing to report.

## Conflicts of Interest

The authors declare no conflicts of interest.

## Supporting information


Data S1.

## Data Availability

The data that support the findings of this study are available on request from the corresponding author. The data are not publicly available due to privacy or ethical restrictions.
