# Supplementary material for: Inhibitory Potential and Binding Thermodynamics of Scyllatoxin‐Based BH3 Domain Mimetics Targeting Repressor BCL2 Proteins
Source: J Mol Recognit. 2025 Feb 4;38(2):e70001. doi: 10.1002/jmr.70001 (PMC11794977; doi:10.1002/jmr.70001)
Supplement: Supplementary file 1 — Data S1. [file JMR-38-e70001-s001.doc]

Supporting Information

**Inhibitory potential and binding thermodynamics of scyllatoxin-based**

**BH3 domain mimetics targeting repressor BCL2 proteins**

**H. A. D. B. Amarasiri^1^, Danushka Arachchige^1^, Matthew J. K. Vince^1,2^ and Justin M. Holub^1,3,4^**

^1^ Department of Chemistry and Biochemistry, Ohio University, Athens, OH 45701, USA; ^2^ Institut für Bioanalytische Chemie, Biotechnologisch-Biomedizinisches Zentrum, Fakultät für Chemie und Mineralogie, Universität Leipzig, Leipzig, Germany; ^3^ Molecular and Cellular Biology Program and

^4^ Edison Biotechnology Institute, Ohio University, Athens, OH 45701, USA.

**Reagents and chemicals.** Fmoc-protected amino acids and PAL-AM resin were purchased from Novabiochem (Billerica, MA). N,N-diisopropylethylamine (DIEA), N-methyl-2-pyrrolidone (NMP), piperidine, triisopropylsilane (TIPS), isopropyl β-D-1-thiogalactopyranoside (IPTG), ammonium persulfate (APS), bovine serum albumin (BSA), acetic anhydride, sodium phosphate monobasic monohydrate, trypsin, sodium phosphate dibasic heptahydrate, 4-methylmorpholine (MMP), and formic acid were obtained from Sigma-Aldrich (St. Louis, MO). 2-(6-chloro-1-H-benzotriazole-1-yl)-1,1,3,3-tetramethylaminium hexafluorophosphate (HCTU) was purchased from Peptides International (Louisville, KY), 5-carboxyfluorescein (5-CF) was obtained from Santa Cruz Biotechnology (Dallas, TX). 2,2,2-trifluoroethanol (TFE) and trifluoroacetic acid (TFA) were purchased from Acros Organics (Morris Plains, NJ). O-(7-azabenzotriazol-1-yl)-N,N,N′,N′-tetramethyluronium hexafluorophosphate (HATU) was purchased from P3 biosystems (Louisville, KY). Tris, sodium chloride, sodium hydroxide, acetic acid, dichloromethane (DCM), bisacrylamide, tetramethylethylenediamine (TEMED), methanol, sodium dodecyl sulfate (SDS), imidazole, LB agar, ampicillin, bacterial protein extraction reagent (B-PER), protease inhibitor cocktail and phenol were purchased from Thermo Scientific (Waltham, MA). Acetonitrile (ACN) was purchased from VWR Analytical (Sunnyvale, CA). Protein marker was obtained from New England Biolabs (Ipswitch, MA). Ni-NTA resin was purchased from Molecular Cloning Laboratories (San Francisco, CA). LB medium was obtained from MP Biomedicals (Santa Ana, CA).


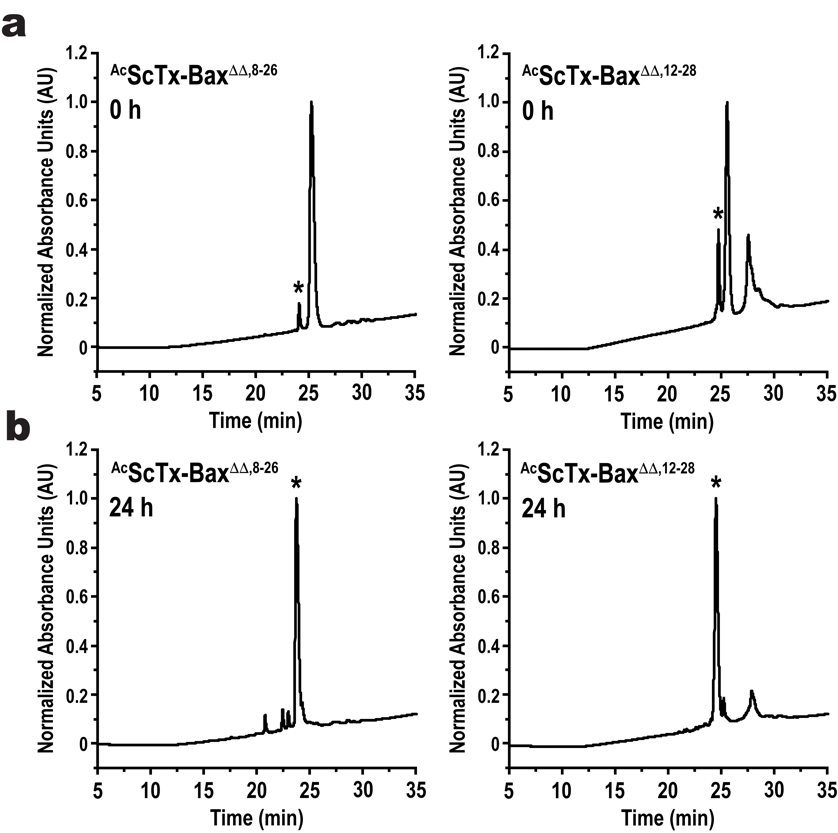


**Figure S1.** Representative reversed-phase HPLC chromatograms of peptide oxidation reactions. Top row (a) shows chromatograms of reduced peptides ^Ac^ScTx-Bax^ΔΔ,8-26^ and ^Ac^ScTx-Bax^ΔΔ,12-28^ prior to oxidation (0 h); bottom row (b) shows chromatograms of oxidized peptides ^Ac^ScTx-Bax^ΔΔ,8-26^ and ^Ac^ScTx-Bax^ΔΔ,12-28^ following the oxidation reaction (24 h). Time of oxidation (h) is shown in each respective chromatogram. Fully-oxidized product peaks are indicated with an asterisk (*). All spectra were monitored at 214 nm and plotted as normalized absorbance units versus time.


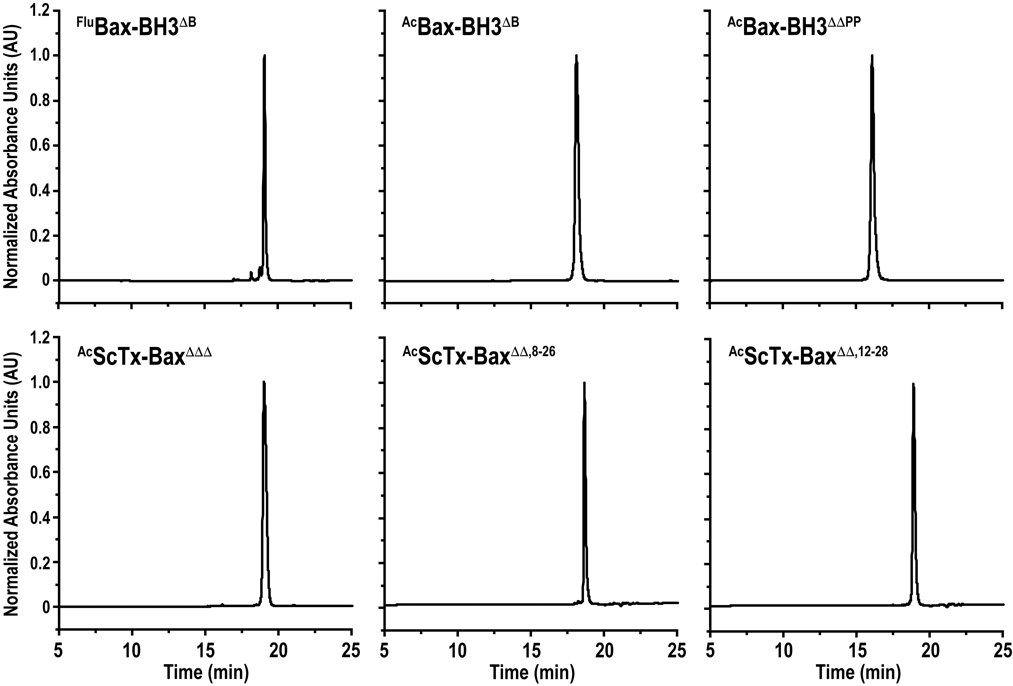


**Figure S2.** Analytical reversed-phase HPLC spectra of peptides used in this work. All spectra were monitored at 214 nm and plotted as normalized absorbance units versus time.


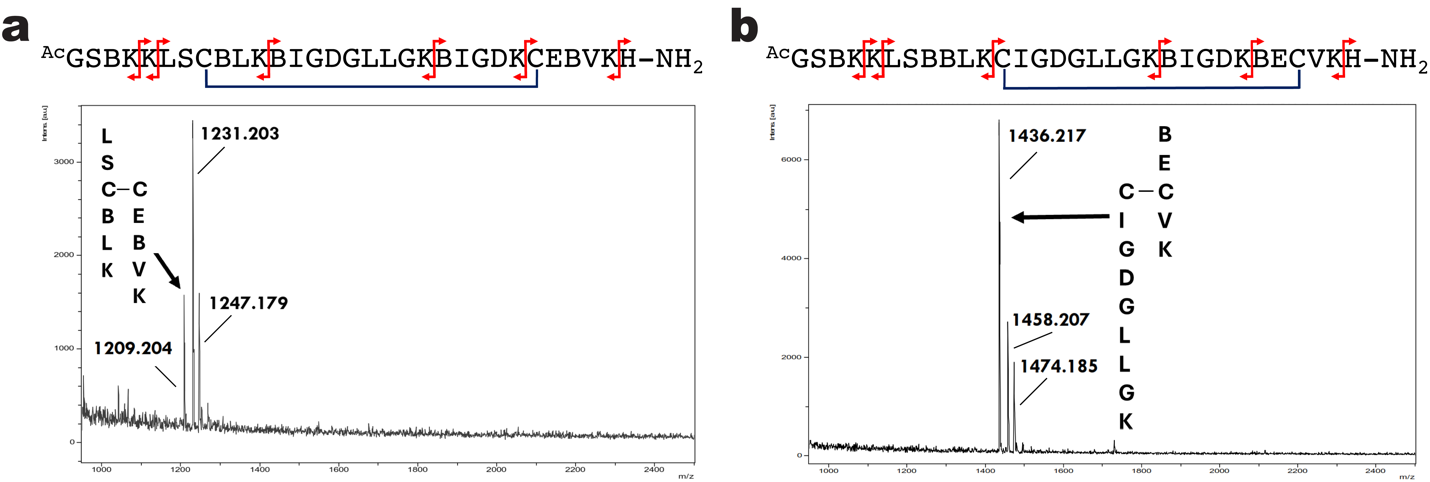


**Figure S3.** Mass spectra of fragments isolated from trypsin digests of oxidized (a) ^Ac^ScTx-Bax^ΔΔ,8-26^ and (b) ^Ac^ScTx-Bax^ΔΔ,12-28^ peptides. Peptide sequences are shown above each spectra. Trypsin cut sites are indicated within each sequence with bidirectional red arrows. Sequences of disulfide-linked peptide fragments are shown adjacent to corresponding mass peaks. Sodium- and potassium-adducted peaks are also labeled.


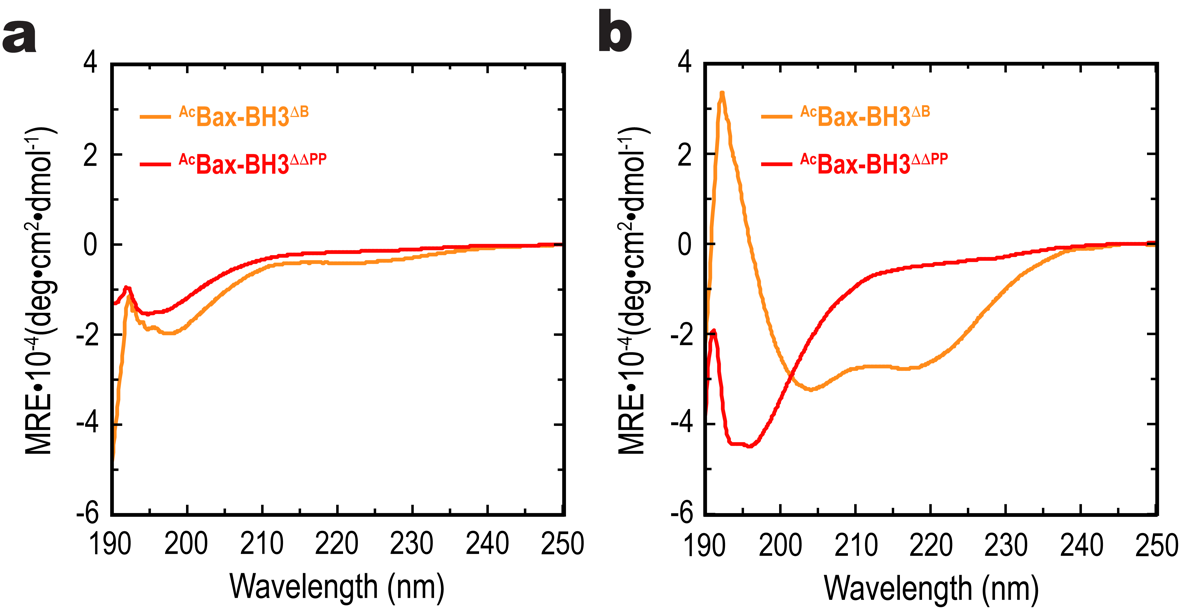


**Figure S4.** Far-UV CD spectra of Bax-BH3 domain peptides (10 μM) in binding buffer (50 mM Tris, 0.1 M NaCl, pH 8) supplemented without (a) or with (b) 30% (v/v) TFE. All solutions were allowed to equilibrate at 20 ºC for 10 min before being analyzed by CD spectropolarimetry.


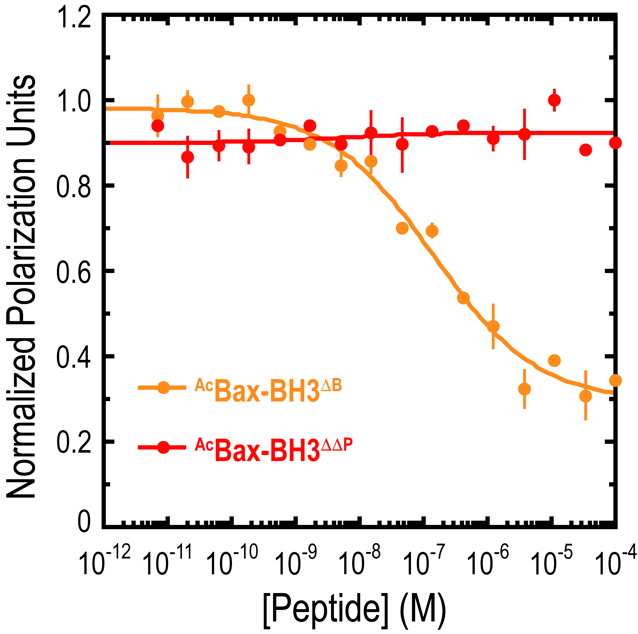


**Figure S5. Results from fluorescence polarization competitive binding assays of acetylated Bax-BH3 domain peptides targeting Bcl-2-ΔTM in binding buffer (50 mM Tris, 0.1 M NaCl, pH 8). Data points represent an average of three independent experiments; error bars are standard deviation. IC_50_ value for ^Ac^Bax-BH3^ΔB^ was 141.0 nM; IC_50_ value for ^Ac^Bax-BH3^ΔΔPP^ was not determined.**

| **Peptide** | **Sequence** | **Reduced** | | **Oxidized** | |
| --- | --- | --- | --- | --- | --- |
|  |  | **Calc. (+m/z)** | **Obs. (+m/z)** | **Calc. (+m/z)** | **Obs. (+m/z)** |
| ^Ac^Bax-BH3^ΔB^ | ^Ac^GSTKKLSEBLKRIGDELDSNM-NH_2_ | 2347.67 | 2347.11 | - | - |
| ^Flu^Bax-BH3^ΔB^ | ^Flu^GSTKKLSEBLKRIGDELDSNM-NH_2_ | 2663.94 | 2663.82 | - | - |
| ^Ac^Bax-BH3^ΔΔPP^ | ^Ac^GSTKKLSPCLKPIGDELDSNM-NH_2_ | 2274.65 | 2273.13 | - | - |
| ^Ac^ScTx-Bax^∆∆∆^ | ^Ac^GSBKKLSBBLKBIGDGLLGKBIGDKBEBVKH-NH_2_ | 3157.75 | 3157.89 | - | - |
| ^Ac^ScTx-Bax^ΔΔ,8-26^ | ^Ac^GSBKKLSCBLKBIGDGLLGKBIGDKCEBVKH-NH_2_ | 3193.82 | 3193.72 | 3191.83 | 3191.64 |
| ^Ac^ScTx-Bax^ΔΔ,12-28^ | ^Ac^GSBKKLSBBLKCIGDGLLGKBIGDKBECVKH-NH_2_ | 3193.82 | 3193.45 | 3191.83 | 3191.50 |

**Table S1.** Sequences and mass data of peptides used in this work. Functional BH3 epitope is shown in red, conserved BH3 aspartic acid is cyan, substituted aminobutyric acids (B) are orange.
